# Quantitative Assessment of Temporomandibular Joint Space in Bruxers: A Cross-Sectional Radiographic Study

**DOI:** 10.1155/ijod/3266073

**Published:** 2025-11-18

**Authors:** Ramnarayan B. K., Preeti Patil, Meenakshi Chowdhary, Savita A. M., Krishnanand P. S., Darshana S.

**Affiliations:** ^1^Department of Oral Medicine and Radiology, Dayananda Sagar College of Dental Sciences, Kumaraswamy Layout, Bangalore 560111, India; ^2^Dr Meenakshi Dhanvantari Dental and Implant Clinic, Ghaziabad, India; ^3^Department of Periodontics, Dayananda Sagar College of Dental Sciences, Kumaraswamy Layout, Bangalore 560111, India; ^4^Department of Oral Pathology, Dayananda Sagar College of Dental Sciences, Kumaraswamy Layout, Bangalore 560111, India

**Keywords:** bruxism, CBCT, condylar positioning, joint space, temporomandibular joint, TMJ disorders

## Abstract

**Background:**

Bruxism is a parafunctional habit that can lead to significant dental and musculoskeletal complications. Chronic bruxism may impose excessive and abnormal mechanical loads on the temporomandibular joint (TMJ), potentially altering joint morphology and joint space dimensions. These changes can be subtle and asymptomatic initially, but they may progress to disorders such as disc displacement, joint pain, and functional limitations. Radiographic evaluation of TMJ space, particularly through advanced imaging modalities such as cone beam computed tomography (CBCT), offers a noninvasive means of quantifying joint space alterations. However, the relationship between bruxism and TMJ space narrowing or asymmetry remains inadequately understood, with inconsistent findings across existing literature.

**Objective:**

To quantitatively evaluate the TMJ space in individuals with bruxism using CBCT and compare findings with matched nonbruxer controls.

**Methods:**

A cross-sectional study was conducted on 75 patients clinically diagnosed with bruxism (age 20–45 years, 36 males, 39 females). CBCT scans were analyzed to measure anterior joint space (AJS), superior joint space (SJS), and posterior joint space (PJS) bilaterally. Data were compared to normative values from 30 age- and sex-matched nonbruxers. Independent *t*-test was used to compare joint space dimensions between bruxers and nonbruxers, with significance set at *p* < 0.05.

**Results:**

Bruxers demonstrated a significantly reduced SJS (2.73 ± 0.39 mm) and increased PJS (3.29 ± 0.44 mm) compared to nonbruxers (3.16 ± 0.34 mm and 2.80 ± 0.37 mm, respectively) on CBCT analysis (*p* < 0.001), indicating distinct condylar displacement patterns. Age was significantly negatively correlated with SJS, indicating progressive joint space narrowing with advancing age in bruxers. Male bruxers showed significantly higher PJS compared to females (*p* = 0.018), potentially reflecting gender-based anatomical or functional variation in response to bruxism.

**Conclusion:**

The present study demonstrated that bruxers exhibit significantly increased posterior and reduced SJS dimensions compared to nonbruxers, indicating early condylar positional alterations detectable on CBCT that may aid in timely diagnosis and prevention of symptomatic temporomandibular joint disorders.

## 1. Introduction

Bruxism is a multifactorial parafunctional habit characterized by the repetitive activity of masticatory muscles, typically manifesting as involuntary teeth grinding or clenching during sleep or wakefulness [1]. It is broadly classified into awake bruxism, which is often linked to stress and anxiety, and sleep bruxism, which is considered a sleep-related movement disorder with neurological and psychological correlations [2]. The etiology of bruxism involves an intricate interplay of psychosocial, genetic, and peripheral factors, and although it may initially present as a benign behavioral pattern, its chronic persistence can lead to significant clinical sequelae [3]. One of the most affected anatomical regions in bruxism is the temporomandibular joint (TMJ), a complex synovial articulation whose function relies on the integrity of its osseous, cartilaginous, and muscular components. Prolonged parafunctional loading on the TMJ can result in condylar remodeling, displacement, or even degenerative changes in the articular structures, ultimately contributing to temporomandibular disorders (TMDs), which are often manifested as joint pain, restricted mouth opening, crepitus, or joint clicking [4]. Quantitative evaluation of TMJ spaces, particularly the anterior joint space (AJS), superior joint space (SJS), and posterior joint space (PJS), provides essential diagnostic insight into condylar position and joint health [5]. Among the available imaging modalities, cone-beam computed tomography (CBCT) offers high-resolution, three-dimensional imaging capabilities with relatively low radiation exposure, making it ideal for evaluating TMJ anatomy with precision [6]. However, current literature lacks comprehensive comparative morphometric data on TMJ joint spaces in individuals with bruxism versus healthy controls, and this gap underscores the need for objective, imaging-based assessment of subclinical joint alterations. The scope of this study lies in utilizing CBCT to detect early structural deviations in TMJ morphology among bruxers, which may otherwise go unnoticed during clinical examination. Such data can support preventive and therapeutic strategies aimed at minimizing the risk of progression to symptomatic TMDs. Therefore, the present study aims to evaluate and compare the TMJ joint space dimensions in individuals with bruxism and healthy nonbruxers using CBCT. Specifically, the objectives are to (1) measure the AJS, SJS, and PJS in bruxers using CBCT, (2) compare these measurements with age- and sex-matched nonbruxers, (3) assess deviations in condylar position in the sagittal plane, and (4) explore the diagnostic implications of altered joint space morphology for early detection of TMJ dysfunction.

## 2. Materials and Methods

The present investigation was structured as an observational cross-sectional study aimed at evaluating the morphometric alterations in TMJ space dimensions associated with bruxism, using CBCT as the primary imaging modality.

The sample size for the study was calculated using the formula:

  $$\begin{array}{c} n={\left[\left(Z_{\alpha /2}+Z_{\beta }\right)÷\delta \right]}^{2}\times 2\sigma^{2}, \end{array}$$

where *Z*_α/2_ = 1.96, (for 95% confidence), *Z*^β^ = 0.84 (for 80% power), σ = 0.8 mm (estimated standard deviation [SD] based on pilot data), and δ = 0.4 mm (minimum expected difference in joint space measurements between groups). Substituting the values, the formula becomes: *n* = 62.72. To enhance statistical power, the final sample size was rounded up to 105 participants.

A total of 105 participants were enrolled and divided into two groups. The bruxism cohort consisted of 75 individuals, including 36 males and 39 females, within the age range of 20–45 years (mean age: 31.7 ± 6.5 years). In parallel, a control group (nonbruxers) comprising 30 healthy, age- and sex-matched subjects with no clinical evidence of bruxism was recruited to ensure valid comparative analysis.

Inclusion criteria for the bruxism group were stringently defined, incorporating only those patients with a clinically confirmed diagnosis of either awake or sleep bruxism, in accordance with the diagnostic standards established by the American Academy of Sleep Medicine (AASM). Subjects with any history of prior TMJ trauma, surgery, or treatment were excluded to eliminate potential confounding variables. An essential prerequisite for inclusion was the availability of high-resolution CBCT scans to facilitate accurate and reproducible measurement of joint spaces.

**Figure 1:**
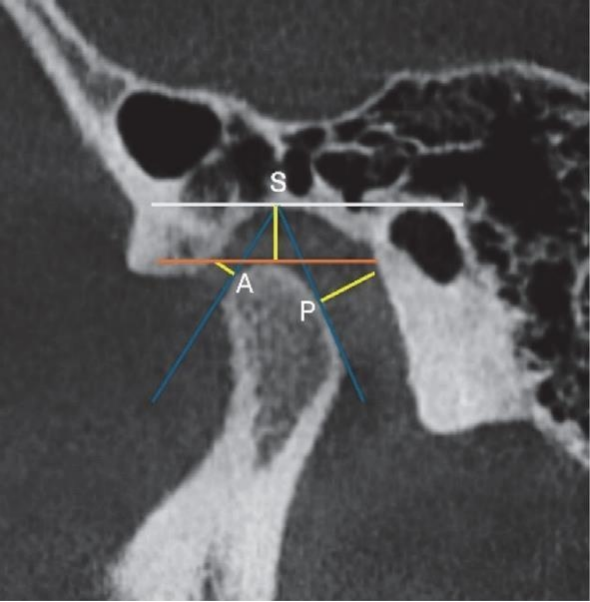
Sagittal section of TMJ in CBCT showing reference points for measurement of AJS, SJS, and PJS.

**Figure 2:**
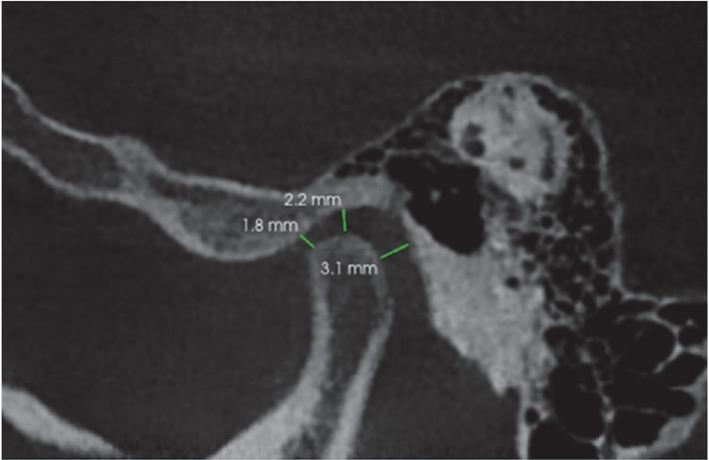
Sagittal section of TMJ in CBCT showing joint space dimensions.

The CBCT imaging protocol was standardized to ensure consistency and precision. CBCT scans were standardized with a voxel size of 0.3 mm and 8 cm × 8 cm field of view (FOV) at mandibular rest position to minimize positional variation of the condyle (as recommended in Alhammadi et al. [7]). CBCT scans were acquired using *(Planmeca ProMax 3D Mid*) at 90 kVp, 10 mA, and 12 s exposure time, and images were analyzed using Romexis *Viewer* to ensure standardized image processing. Scans were acquired with a voxel resolution of 0.3 mm and a FOV measuring 8 cm × 8 cm, parameters deemed optimal for the detailed visualization of the TMJ's bony anatomy. Morphometric analysis was performed at the mandibular rest position, with particular focus on the AJS, SJS, and PJS as follows. First, on the axial sections, slices that showed the widest part of the condyle were selected. True sagittal views with a thickness of 2 mm were created using this slice an the central most sagittal section was taken for measurements of the anterior, superior, and PJSs. The following steps were followed to measure joint space. A horizontal line along the top edge of the glenoid fossa was drawn (white line). The point where this line touched the glenoid fossa was taken as reference point (S). Then, from this point, lines were drawn to the most prominent points on the front (A) and back (P) of the condyle. Another horizontal line was drawn touching the superior most point on the condyle (orange line) (Figure 1). Finally, the shortests distance from point A and P straight up to the glenoid fossa were measured that depicted anterior (AJS) and PJS and line from point A to the superior-most point on the condyle was taken as SJS (Figure 2). These are critical indices that reflect the spatial orientation and functional integrity of the condyle within the glenoid fossa.

To quantify and compare the joint space measurements between bruxers and nonbruxers, comprehensive statistical analysis was conducted. Descriptive statistics, including the mean and SD, were computed for each of the three joint space dimensions across both groups. Group differences were evaluated using an independent *t*-test, a robust parametric method suitable for assessing mean variations between two independent samples. The threshold for statistical significance was set at *p* < 0.05, thereby ensuring that only results with high confidence levels were considered meaningful. This methodological framework was designed to yield reliable, clinically translatable insights into the biomechanical repercussions of bruxism on the TMJ complex.

### 2.1. Ethical Considerations

This is an observational study, and no direct patient identifiers or sensitive data were disclosed. All ethical guidelines were adhered to. As the CBCT data was retrospectively obtained from collaborating private dental clinics for the purpose of academic analysis and literature support, an informed consent waiver and ethical clearance waiver were granted by the institutional ethics committee.

## 3. Results

The present cross-sectional radiographic study evaluated the TMJ space dimensions—specifically the AJS, SJS, and PJS—in two groups: bruxers (*n* = 75) and a control group (*n* = 30). The measurements were obtained bilaterally and expressed as mean values with SD.

### 3.1. TMJ Joint Space in Nonbruxers

As shown in Table 1, among nonbruxers, the mean AJS was 2.28 ± 0.30 mm on the right and 2.30 ± 0.32 mm on the left, with an overall mean of 2.29 ± 0.31 mm. The SJS showed an overall mean of 3.16 ± 0.34 mm, with 3.15 ± 0.35 mm on the right and 3.17 ± 0.33 mm on the left. The PJS a mean of 2.81 ± 0.36 mm on the right, 2.79 ± 0.38 mm on the left, and an overall mean of 2.80 ± 0.37 mm.

### 3.2. TMJ Joint Space in Bruxers

As shown in Table 2, the mean AJS was 2.12 ± 0.35 mm on the right and 2.16 ± 0.37 mm on the left, with an overall mean of 2.14 ± 0.36 mm. The SJS showed an overall mean of 2.73 ± 0.39 mm, being 2.71 ± 0.41 mm on the right and 2.75 ± 0.38 mm on the left. The PJS showed the highest values, with a mean of 3.30 ± 0.43 mm on the right, 3.28 ± 0.45 mm on the left, and an overall mean of 3.29 ± 0.44 mm.

### 3.3. Comparison With Control Group

As outlined in Table 3, the control group had a higher SJS (mean = 3.16 ± 0.34 mm) compared to the bruxers (mean = 2.73 ± 0.39 mm), and this difference was statistically significant (*p*  < 0.001). Conversely, the PJS was significantly increased in bruxers (mean = 3.29 ± 0.44 mm) compared to controls (mean = 2.80 ± 0.37 mm) (*p*  < 0.001). The AJS showed no statistically significant difference between the bruxers (mean = 2.14 ± 0.36 mm) and the control group (mean = 2.29 ± 0.31 mm) (*p* = 0.054).

### 3.4. Correlation Between Demographics and Joint Space in Bruxers

A significant negative correlation was observed between age and SJS in bruxers (*r = −*0.312, *p* = 0.006), indicating that older bruxers tend to have reduced SJS values (Table 4). Additionally, gender-wise comparison showed that male bruxers (*n* = 34) had a significantly higher PJS (mean = 3.41 ± 0.39 mm) compared to female bruxers (*n* = 41) (mean = 3.19 ± 0.46 mm), with a *p*-value of 0.018, suggesting a gender-based variation in condylar positioning under bruxism-induced stress (Table 5)

## 4. Discussion

Bruxism, particularly in its sleep-related form, has garnered increasing attention due to its potential impact on the TMJ structures. According to Maluly et al. [8], the prevalence of sleep bruxism in the general population is estimated to be substantial when assessed through objective polysomnographic techniques. Beyond its mechanical effects, bruxism is frequently linked to psychological factors such as anxiety and depression, further complicating the clinical presentation and diagnosis [9].

Radiographic and imaging advancements have allowed for a more detailed evaluation of TMJ alterations associated with parafunctional habits. Magnetic resonance imaging (MRI) remains the gold standard for assessing soft tissue changes, including disc displacement, and shows a reliable correlation with clinical findings in patients with TMJ dysfunction [10]. Additionally, CBCT has been widely validated as a highly effective modality for assessing bony morphology and joint space asymmetries in TMJ evaluations [11].

Given this context, our study sought to quantify the changes in joint space dimensions in bruxers using CBCT and explore any associations with demographic variables. These imaging findings not only aid in early diagnosis and management but also contribute to understanding the adaptive and degenerative joint responses in chronic bruxers.

The present study quantitatively assessed TMJ space variations—specifically AJS, SJS, and PJS—among nonbruxers (Table 1) and bruxers (Table 2) using CBCT. The results indicated statistically significant alterations in joint space measurements among bruxers. Notably, SJS was significantly reduced (mean 2.73 ± 0.39 mm) and PJS was significantly increased (mean 3.29 ± 0.44 mm) in the bruxer group compared to controls, while the AJS difference was statistically nonsignificant (*p* = 0.054) (Table 3). These findings reinforce the hypothesis that bruxism contributes to mechanical loading and remodeling of the condylar-fossa relationship. Maluly et al. [8] have reported that sleep bruxism is prevalent in approximately 8%–15% of the general population, with repetitive parafunctional activity being a key risk factor for TMJ stress and dysfunction. The observed asymmetry and PJS widening may be attributed to repetitive clenching and grinding forces that can shift the condyle anteriorly within the glenoid fossa, increasing the PJS. Michael Radu et al. [12] conducted a study on effect of clenching on condylar position and found that the translation force of clenching displaced the condyle into the anterior–superior equilibrium position. This explains decreased SJS and increased PJS in bruxism patients. The results of the current study are in alignment with these findings.

Furthermore, psychological comorbidities such as anxiety and depression, which are commonly associated with bruxism, can influence masticatory muscle hyperactivity, thereby altering TMJ biomechanics [9]. These psychosomatic factors likely contribute to microtrauma and remodeling changes in the joint spaces, especially evident in the posterior and superior compartments.

In a CBCT study, Barghan et al. [11] highlighted the utility of CBCT in detecting bony adaptations, including flattening and joint space narrowing, in TMJ disorders. Our study aligns with their conclusions, showing that bruxers exhibit measurable dimensional differences even in the absence of overt clinical symptoms.

Additionally, Giozet et al. [13] found that structural changes such as disc displacement with reduction were common in chronic TMJ patients and correlated well with imaging finding. The morphological findings also resonate with Yamada et al. [14], who reported condylar bony changes in TMD patients with self-reported parafunctional habits, underscoring the association between behavior and structural TMJ alterations.

In this study, bruxers demonstrated a significantly reduced SJS (mean 2.73 ± 0.39 mm) and a significantly increased PJS (mean 3.29 ± 0.44 mm) compared to controls, while the AJS difference did not reach statistical significance. Additionally, advancing age in bruxers correlated negatively with SJS (*r* = –0.312, *p* = 0.006), and male bruxers exhibited larger PJS than females (mean 3.41 ± 0.39 mm vs. 3.19 ± 0.46 mm, *p* = 0.018) (Table 4). These findings can be contextualized in light of existing literature on sleep bruxism prevalence, psychological comorbidities, and imaging-based TMJ assessments.

Bruxism imposes repetitive mechanical loading on the TMJ, which in turn may lead to adaptive condylar repositioning. Maluly et al. [8] reported that sleep bruxism affects up to 8%–15% of adults when diagnosed polysomnographically, underscoring the clinical relevance of even subclinical joint changes in a sizable population. Our observation of condylar repositioning (increased PJS) is consistent with chronic masticatory hyperactivity that may displace the condyle anteriorly due to the strong pull of the lateral pterygoid under load. In contrast, healthy controls maintain a more balanced joint-space distribution, as illustrated by their higher SJS (3.16 ± 0.34 mm) and lower PJS (2.80 ± 0.37 mm).

Psychological factors such as anxiety and depression are known to exacerbate bruxism intensity, thereby amplifying TMJ stress [3]. Gungormus and Erciyas demonstrated a positive association between bruxism and elevated anxiety/depression scores, suggesting that psychosomatic drivers can potentiate occlusal force generation. In our cohort, older bruxers exhibited further SJS narrowing—likely reflecting cumulative microtrauma and gradual condylar remodeling under chronic parafunctional loads [9]. This age-related joint space constriction parallels histologic evidence of degenerative disc changes reported by Cui et al. [10], wherein chronic inflammation degrades collagen fibrils in the TMJ disc, promoting anterior disc thinning and reducing superior space.

CBCT has proven invaluable for assessing condylar morphology and joint-space asymmetries. Barghan et al. highlighted CBCT's superior ability to delineate bony adaptations—such as subcortical sclerosis or mandibular condyle flattening—in TMD patients compared to conventional radiography [11]. Our use of CBCT allowed precise quantification of SJS and PJS differences; the significant SJS reduction (2.73 ± 0.39 mm vs. 3.16 ± 0.34 mm in controls, *p*  < 0.001) likely reflects early bony remodeling and adaptive changes in the glenoid fossa margin, consistent with CBCT findings by Yamada et al. [14] in orthognathic patients with parafunctional habits.

The gender-based variation in PJS—greater in male bruxers—may reflect inherent differences in condylar size or musculature. Imanimoghaddam et al. reported that male TMD patients often exhibit larger condylar volumes and distinct condylar positioning patterns on CBCT compared to females [15]. Our data (PJS_M = 3.41 ± 0.39 mm vs. PJS_F = 3.19 ± 0.46 mm, *p* = 0.018) (Table 5) align with this observation, suggesting that male condyles under chronic bruxism stresses may displace further compared to females. This positional shift could predispose males to early degenerative bony changes, as outlined by Milam and Schmitz's molecular investigations into TMJ arthritis development [16].

Comparatively, age-related joint space narrowing is a recognized phenomenon in degenerative TMD. Yamada et al. found that condylar bony changes correlate with self-reported parafunctional habits, noting that older individuals with long-standing bruxism exhibited reduced SJS and early condylar flattening [14]. Our negative correlation between age and SJS (*r* = –0.312, *p* = 0.006) supports this, suggesting a progressive decline in superior joint height that may eventually culminate in clinical dysfunction.

This study's CBCT-based findings bolster the concept that bruxism involves repetitive clenching and grinding forces that can shift the condyle anteriorly within the glenoid fossa. Ikeda and Kawamura demonstrated that anterior condylar positioning on CBCT correlates with increased PJS and reduced SJS [5].

Integrating these radiographic insights with psychosocial and molecular data from prior research highlights the multifactorial nature of TMJ alterations in bruxers. Early identification of such joint space adaptations via CBCT can inform timely interventions, potentially mitigating progression toward irreversible osteoarthritic changes.

The clinical and managerial applications of this study's findings are both multifaceted and far-reaching, offering significant implications for diagnostic refinement and therapeutic strategizing in TMJ dysfunctions associated with bruxism. The precise quantification of joint space alterations through CBCT empowers clinicians with a nuanced understanding of subclinical changes that may precede overt TMDs, thereby facilitating preemptive intervention and mitigating long-term morbidity. From a clinical standpoint, incorporating CBCT-based joint space assessment into routine diagnostic protocols allows for the early identification of condylar displacement, asymmetry, or remodeling that may otherwise remain undetected through conventional two-dimensional imaging modalities or symptomatic evaluation alone. This advanced morphometric insight may be especially vital in treatment planning for prosthodontic rehabilitation, orthodontic alignment, or occlusal splint therapy, where condylar positioning serves as a foundational anatomical reference.

On a managerial and operational level, the integration of standardized CBCT evaluation protocols could enhance interdisciplinary collaboration between general dentists, oral radiologists, and TMJ specialists, thereby fostering a more cohesive and evidence-based model of care. Proposed guidelines emerging from this research advocate for the routine inclusion of CBCT joint space analysis in high-risk bruxism populations—particularly those with parafunctional habits, myofascial discomfort, or occlusal wear facets. These guidelines underscore the necessity for early detection frameworks, structured referral systems, and longitudinal monitoring pathways that transcend reactive management and embrace preventive paradigms. Ultimately, by bridging the gap between structural imaging and functional symptomatology, this study lays the groundwork for a more informed, proactive, and anatomically grounded approach to TMJ health in bruxism patients.

Bruxism is a multifactorial condition strongly associated with TMJ disorders, often manifesting as condylar positional changes and altered joint loading [1, 2]. Ohlmann et al. [17] highlighted a correlation between sleep bruxism and various TMDs, emphasizing that bruxism can contribute to altered loading on the TMJ and surrounding musculature. However, the exact impact on condylar displacement is not consistently demonstrated across studies. According to Commisso et al. [18] prolonged clenching forces associated with awake bruxism exert greater shear stress on TMJ structures compared to cyclic forces in sleep bruxism, potentially leading to morphological alterations such as condylar flattening, disc deformation, and joint surface remodeling. Epidemiological studies indicate a considerable prevalence of bruxism among adults, reinforcing its clinical importance as a risk factor for TMJ dysfunction [3]. The present study provides morphometric evidence that bruxers exhibit increased posterior and reduced SJS dimensions which is in contrast to some of the existing literature, reflecting early condylar displacement consistent with previous findings on the biomechanical repercussions of abnormal loading [4]. Nevertheless, more robust longitudinal data are required to delineate the precise effects of bruxism on condylar remodeling and occlusion stability.

Bruxism can significantly affect occlusion, not only through tooth wear and loss of vertical dimension, but also via altered mechanics, muscle activity, and dental-restorative consequences. Chronic clenching and grinding can lead to teeth wear, ename attirion, and flattening of occlusal surfaces potetntailly, altering the intercuspation of teeth [1, 3, 4]. Bruxism can lead to muscle hyperativity leading to bite instability and uneven force distribution. Psychosomatic factors such as anxiety and depression exacerbate these occlusal changes [8, 9] Repetitive parafunctional forces may displace the condyle anteriorly and superiorly, which can shift the mandibular position and modify the occlusal scheme.

Though the existing literature does give an insight into the effect of bruxism on condylar displacement and on occlusion, more literature and studies are required to specifically answer two clinical questions (1) what is the long-term impact of bruxism on condylar displacement patterns, and (2) how does bruxism influence occlusal changes over time?

While CBCT offers excellent spatial resolution for osseous evaluation, future studies incorporating MRI may provide deeper insights into the soft tissue and discal adaptations within the TMJ, thereby complementing CBCT findings and strengthening diagnostic evidence [5].

## 5. Conclusion

The present study demonstrated that bruxers exhibit significantly increased posterior and reduced SJS dimensions compared to nonbruxers, indicating early condylar positional alterations detectable on CBCT that may aid in timely diagnosis and prevention of symptomatic TMD. These findings also highlight the diagnostic value of CBCT in detecting subclinical TMJ alterations.

### 5.1. Limitation

The cross-sectional design restricts the ability to establish causal relationships between bruxism and TMJ space alterations.

### 5.2. Future Perspective

Longitudinal studies with larger cohorts and functional imaging integration are warranted to explore progressive TMJ remodeling and therapeutic outcomes in bruxism patients. Future studies incorporating both CBCT and MRI could further elucidate the interplay between hard and soft tissue changes in bruxism-related TMJ disorders.

## Figures and Tables

**Table 1:** TMJ joint space in nonbruxers (*n* = 30).

Joint space	Right side (mm)	Left side (mm)	Overall mean ± SD (mm)
AJS (anterior joint space)	2.28 ± 0.30	2.30 ± 0.32	2.29 ± 0.31
SJS (superior joint space)	3.15 ± 0.35	3.17 ± 0.33	3.16 ± 0.34
PJS (posterior joint space)	2.81 ± 0.36	2.79 ± 0.38	2.80 ± 0.37

**Table 2:** Mean TMJ joint space measurements in bruxers (*n* = 75).

Joint space	Right side (mm)	Left side (mm)	Overall mean ± SD (mm)
AJS	2.12 ± 0.35	2.16 ± 0.37	2.14 ± 0.36
SJS	2.71 ± 0.41	2.75 ± 0.38	2.73 ± 0.39
PJS	3.30 ± 0.43	3.28 ± 0.45	3.29 ± 0.44

**Table 3:** Statistical comparison of TMJ space in nonbruxers and bruxers.

Joint space	Control group mean ± SD (mm)	Bruxers mean ± SD (mm)	*p*-Value	Statistical significance
AJS	2.29 ± 0.31	2.14 ± 0.36	0.054	Not significant
SJS	3.16 ± 0.34	2.73 ± 0.39	<0.001	Significant ↓ in bruxers
PJS	2.80 ± 0.37	3.29 ± 0.44	<0.001	Significant ↑ in bruxers

**Table 4:** Correlation of age with TMJ joint space in bruxers (*n* = 75).

Demographic variable	Joint space	Mean ± SD (mm)	Correlation coefficient (*r*)/test statistic	*p*-Value	Interpretation
Age (years)	AJS	2.14 ± 0.36	*r* = –0.105	0.346	No significant correlation
	SJS	2.73 ± 0.39	*r* = –0.312	0.006	Significant negative correlation
	PJS	3.29 ± 0.44	*r* = +0.124	0.262	Not significant

**Table 5:** Correlation of gender with TMJ Joint space in bruxers (*n* = 75).

Joint space	Male (*n* = 34)	Female (*n*-41)	Independent sample *t* test	*p*-Value	Interpretation
AJS	2.19 ± 0.34	2.10 ± 0.38	*t* = 1.123	0.265	Not significant
SJS	2.78 ± 0.35	2.68 ± 0.42	*t* = 1.022	0.310	Not significant
PJS	3.41 ± 0.39	3.19 ± 0.46	*t* = 2.420	0.018	Significant difference (↑ in males)

## Data Availability

The data that support the findings of this study are available from the corresponding author upon reasonable request.
